# Safety of Carotid Endarterectomy for Symptomatic Stenosis by Age: Meta-Analysis With Individual Patient Data

**DOI:** 10.1161/STROKEAHA.122.040819

**Published:** 2023-01-24

**Authors:** Ya Yuan Rachel Leung, Kasia Bera, Daniel Urriza Rodriguez, Alan Dardik, Jean-Louis Mas, Gioele Simonte, Kittipan Rerkasem, Dominic P.J. Howard

**Affiliations:** 1 Wolfson Centre for Prevention of Stroke and Dementia, Nuffield Department of Clinical Neurosciences, University of Oxford, UK (Y.Y.R.L., D.P.J.H.).; 2 Department of Vascular Surgery, Oxford University Hospitals NHS Trust, UK (K.B., D.U.R., D.P.J.H.).; 3 Yale Department of Surgery, Departments of Surgery and Cellular and Molecular Physiology, Yale School of Medicine, New Haven, CT (A.D.).; 4 Department of Surgery, VA Connecticut Healthcare System, West Haven (A.D.).; 5 Department of Neurology, GHU Paris, Hôpital Sainte-Anne, Université Paris-Cité, Inserm, France (J.-L.M.).; 6 Vascular and Endovascular Surgery Unit, Santa Maria della Misericordia, University of Perugia, Italy (G.S.).; 7 Environmental - Occupational Health Sciences and Non-Communicable Diseases Research Group, Research Institute for Health Sciences, Chiang Mai University, Thailand (K.R.).; 8 Clinical Surgical Research Center, Department of Surgery, Faculty of Medicine, Chiang Mai University, Thailand (K.R.).

**Keywords:** aging, carotid endarterectomy, carotid stenosis, ischemic stroke, morbidity

## Abstract

There is uncertainty whether elderly patients with symptomatic carotid stenosis have higher rates of adverse events following carotid endarterectomy. In trials, recurrent stroke risk on medical therapy alone increased with age, whereas operative stroke risk was not related. Few octogenarians were included in trials and there has been no systematic analysis of all study types. We aimed to evaluate the safety of carotid endarterectomy in symptomatic elderly patients, particularly in octogenarians.

**Methods:**

We did a systematic review and meta-analysis of studies (from January 1, 1980 through March 1, 2022) reporting post carotid endarterectomy risk of stroke, myocardial infarction, and death in patients with symptomatic carotid stenosis. We included observational studies and interventional arms of randomized trials if the outcome rates (or the raw data to calculate these) were provided. Individual patient data from 4 prospective cohorts enabled multivariate analysis.

**Results:**

Of 47 studies (107 587 patients), risk of perioperative stroke was 2.04% (1.94–2.14) in octogenarians (390 strokes/19 101 patients) and 1.85% (1.75–1.95) in nonoctogenarians (1395/75 537); *P*=0.046. Perioperative death was 1.09% (0.94–1.25) in octogenarians (203/18 702) and 0.53% (0.48–0.59) in nonoctogenarians (392/73 327); *P*<0.001. Per 5-year age increment, a linear increase in perioperative stroke, myocardial infarction, and death were observed; *P*=0.04 to 0.002. However, during the last 3 decades, perioperative stroke±death has declined significantly in octogenarians (7.78% [5.58–10.55] before year 2000 to 2.80% [2.56–3.04] after 2010); *P*<0.001. In Individual patient data multivariate-analysis (5111 patients), age ≥85 years was independently associated with perioperative stroke (*P*<0.001) and death (*P*=0.005). Yet, survival was similar for octogenarians versus nonoctogenarians at 1-year (95.0% [93.2–96.5] versus 97.5% [96.4–98.6]; *P*=0.08), as was 5-year stroke risk (11.93% [9.98–14.16]) versus 12.78% [11.65–13.61]; *P*=0.24).

**Conclusions:**

We found a modest increase in perioperative risk with age in symptomatic patients undergoing carotid endarterectomy. As stroke risk increases with age when on medical therapy alone, our findings support selective urgent intervention in symptomatic elderly patients.

Carotid endarterectomy (CEA) has been shown to reduce the risk of ischemic stroke in patients with significant recent symptomatic carotid stenosis.^1,2^ However, CEA is associated with perioperative morbidity, including stroke, myocardial infarction (MI), and death, which may offset benefit in high-risk patients. Several clinical characteristics, including age, may be associated with increased perioperative risk,^3,4^ but there has been no systematic analysis of risk associations in symptomatic patients. Determining the risks associated with surgical intervention for symptomatic elderly patients is of great clinical importance as they now represent a significant proportion of patients undergoing CEA in routine clinical practice, and they were largely excluded from previous clinical trials.

Analyses of randomized trials have shown that for patients with symptomatic carotid stenosis on medical therapy alone, recurrent ipsilateral carotid territory stroke rates increase significantly with age; approximately double in patients >75 years (5-year risk of 30.5% [95% CI, 22.5–38.5]) versus <65 years (5-year risk of 18.0% [95% CI, 14.6–21.3]),^5^ whereas operative risks appear to be unrelated.^5–9^ However, even the largest trials have very few outcome events with limited follow-up, and few elderly patients were recruited; therefore, precise estimates of risk, stratified by age, have not been feasible and age-related recruitment bias may undermine any age-risk association.

Observational studies may offer better representative data of procedural outcomes in relation to age; however, individual studies often report conflicting results, and previous systematic reviews of observational studies have included both asymptomatic and symptomatic patients with no individual patient data (IPD) available for multivariate analysis.^10–15^ Very elderly patients seldom require surgical intervention for asymptomatic carotid disease and there is limited evidence to support this. However, those with symptomatic disease often warrant urgent intervention and they reflect real-life practice concerns due to their increased burden of comorbidity, frailty, and cognitive decline. We aimed to determine the safety of CEA for symptomatic patients by age, particularly for octogenarians, by performing the first systematic review of all study types, together with IPD meta-analysis. We included observational studies, registries, and trial cohorts considering potentially confounding factors, including the presence of cardiovascular risk factors, sex, and type of index cerebrovascular event.

## Methods

The data that support the findings of this study are available from the corresponding author upon reasonable request. As this study is a meta-analysis without need for patient consent, institutional review board approval is not required.

### Search Strategy and Selection Criteria

We did a systematic review and meta-analysis (PROSPERO ID: CRD42021285266) of studies reporting the post-CEA stroke risk in patients with symptomatic carotid stenosis, with reference to the participant’s age. We also performed IPD meta-analysis on 4 included studies.

We followed PRISMA (Preferred Reporting Items for Systematic Reviews and Meta-Analyses) and MOOSE (Meta-Analysis of Observational Studies in Epidemiology) reporting guidelines (Table S1 and Figure S1).^16^ We searched MEDLINE, Embase, and the Cochrane Central Register of Controlled Trials (from inception to February 1, 2022) to identify relevant studies of prognosis. We included both observational cohort studies and the CEA treatment groups of randomized trials. Studies that included over 50 patients and provided stroke rates (or the raw data to calculate these) were eligible for inclusion, irrespective of language. A minimum of 50 study participants for inclusion was deemed appropriate in order to allow for comparisons between age groups, and to avoid inclusion of case series, which are prone to significant selection and outcome biases.

In electronic database searches, we used combinations of search terms for carotid stenosis, relevant carotid interventions, and outcome (List S1). We reviewed the bibliographies of all articles identified and relevant systematic and narrative reviews. In cases of multiple publications from the same cohort, the first published study reporting the required data was used, unless the number of patients increased in a later publication, in which case the study reporting the larger cohort was used.

Two reviewers (Y.Y.R.L. and K.B.) independently searched, screened, and selected all studies. Full text of abstracts considered potentially relevant by any reviewer were retrieved. Studies reporting mixed cohorts of patients (with symptomatic and asymptomatic carotid stenosis) were included only if results were stratified by symptom status. Discrepancies were resolved by consensus with a third investigator (D.P.J.H.). The agreement between these observers in their independent assessments of study eligibility of was high (90%; kappa=0.102; SE: 0.04; *P*<0.001).

Y.Y.R.L. extracted data from each study into electronic tables, including study design, funding, setting, population characteristics, index event of symptomatic stenosis, and operation setting. For each outcome of interest, we recorded the outcome definitions, baseline screening methods, follow-up frequency and duration, and outcome ascertainment methods. We assessed the risk of bias and the quality of reporting of individual studies with STROBE (Strengthening the Reporting of Observational Studies in Epidemiology) and ROB2 (A Revised Cochrane Risk-of-Bias Tool for Randomized Trials) criteria.

### Individual Patient Data

All corresponding authors of included studies were contacted to obtain the IPD for multivariate analysis to investigate risk factors associated with postoperative morbidities. Four IPD from Umbria, Italy, the UK National Vascular Registry, EVA-3S trial (Endarterectomy Versus Angioplasty in Patients with Symptomatic Severe Carotid Stenosis), and the Connecticut Hospital Association Chime Data Program were obtained. Principal investigators of these studies coauthor the present report and shared anonymized data on each randomized patient. Baseline data on age, sex, and medical history included the index events of symptomatic stenosis, assessed by local criteria were recorded. Follow-up data included 30-day outcome, the start and end dates of follow-up (or date of death), and nonprocedural stroke incidence, etiology, and severity. To identify factors associated with post-CEA morbidities, pooled odds ratio (OR) from demographic variables, vascular risk factors, and index event of symptomatic stenosis were calculated for patients in the IPD, with logistics regression. IPD were reconstructed from 6 included studies, which provided Kaplan-Meier survival curves or crude stroke and death rates.^17^

### Statistical Analysis: IPD and Meta-Analysis

The primary outcome was any stroke or death during the periprocedural period (defined as up to 30-days postoperation) and until the end of study follow-up. We calculated the average annual stroke rate as the number of strokes per 100 person-years of observation. If the total patient-years of observation was not reported, we calculated this as the number of patients multiplied by the mean follow-up time, excluding studies reported only periprocedural outcomes. Pooled risk estimates were obtained by Mantel-Haenszel method, with 95% CIs calculated to allow for extrabinomial variation, since standard methods of calculating CIs can produce artificially narrow intervals if there is heterogeneity of risk between different studies.

The heterogeneity of risk across studies was assessed with I² tests. For the IPD analysis, we assessed for heterogeneity and clustering using I² tests and Egger funnel plot analysis. We performed both 2-stage and 1-stage IPD analysis to fully adjust for possible clustering. As the results of both approaches were consistent, we included the pooled IPD analysis results in the article. Stroke was defined as focal neurological deficit lasting longer than 24 hours in any cerebrovascular territory, and death was defined as all-cause mortality. We defined older patients as ≥80 years-old, and younger patients as <80 years-old. In 5-years age-stratified analysis, <65 years of age was used as the reference group for comparisons with any older age groups (ie, age 65–69, 70–74, 75–79, ≥80 years).

For each study that provided age group results for comparisons, we calculated OR and 95% CI for the risk of stroke, MI, and death during follow-up for younger and older patients (separately for age cut-off at 65, 70, 75, and 80 years), and combined estimates by fixed-effects meta-analysis (Mantel-Haenszel-Peto method) when there was limited heterogeneity between studies (I² test *P*<0.05). Otherwise, we performed random-effects analysis. The pooled analysis was primarily stratified by the patients’ age ≥80 years (referred as older) versus <80 years (referred as younger). We performed pooled analysis of annual stroke rate and annual death rate in older versus younger patients stratified by year of publication (by decade), and medical history including diabetes, hyperlipidaemia, and coronary artery disease.

Cumulative incidence of stroke and death post-CEA were calculated from longitudinal studies. Six studies provided Kaplan-Meier curves of stroke rates and survival. This enabled extraction of patient-level data for survival analysis. Stroke and death incidence classified by 5-year age groups (<65 years, 65–69 years, 70–74 years, 75–79 years, and ≥80 years) was calculated from studies reporting age-stratified stroke and death incidence and the IPD. Linear regression was used to estimate the additional perioperative risks per 5-year increase in age. Studies that provided older versus younger age group-specific incidence are included in the meta-analysis. Group differences in continuous parametric variables (such as age) were examined with Student *t* test or 1-way ANOVA, as appropriate. Group differences in categorical variables were examined with Fisher exact test or χ2 test, as appropriate. *P*<0.05 were considered significant.

In the event of heterogeneity between studies in the association of age with stroke risks, further sensitivity analyses were based on year of study (by decade), study type, and grading of study quality based on STROBE criteria and key criteria required for meta-analysis, which includes the following: study objectives stated clearly; patient selection criteria stated clearly; patients enrolled consecutively without predetermined selection; interventions adequately described; outcome definitions provided; rate of dropout or crossover to endarterectomy or stenting of <20%; and outcome ascertainment by a neurologist. We graded each study as low, medium, or high based on these criteria. Potential publication bias in relation to the association of stenosis with stroke risk was assessed with an Egger funnel plot. Analysis was performed using R version 4.1.1 and Review Manager (RevMan, computer program, version 5.4, The Cochrane Collaboration, 2020).

## Results

The search of electronic published work produced 8726 references (including 986 duplicates), of which 39 articles were eligible for inclusion (Figure S1). A total of 32 articles were further identified by hand-searching of reference lists, 8 of which were eligible. Forty-seven studies, comprising 107 587 patients and 23 188 octogenarians, met the inclusion criteria: 7 prospective cohort studies, 25 retrospective cohort studies, 6 registries, and 7 randomized controlled trials.^1,6,7,12,18–58^ Most of the cohorts were from North America (n=16), Europe (n=11), with 3 from Asia, 1 from Australia, and 1 from New Zealand. The year of publication, demographic details, study design, and surgical methods are summarized in Table S2.

**Figure 1.**
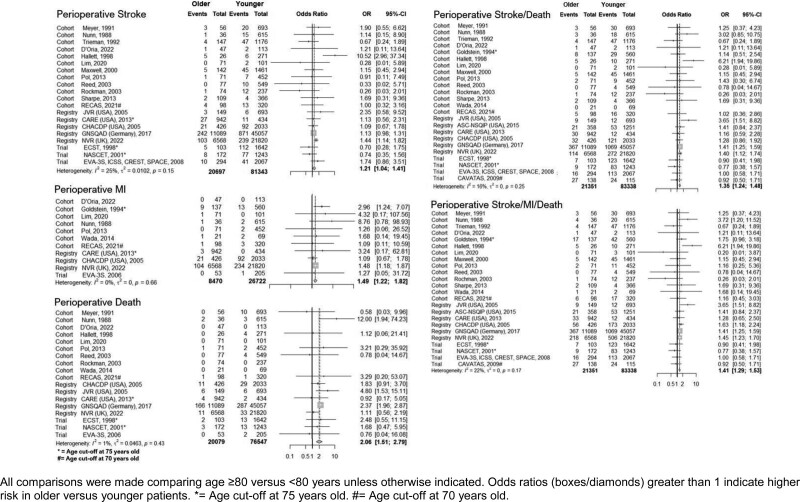
Meta-analysis of perioperative any stroke, myocardial infarction (MI), and death.

Data from 104 689 patients, including 21 115 octogenarians, were included in the meta-analysis of perioperative stroke, MI, and death. Older versus younger patients had higher risk of stroke, MI, and death via in-study cut-point analysis (Figure 1; Figure S6). Risk of perioperative stroke was 1.89% (95% CI, 1.79–1.95%) overall, and 2.04% (95% CI, 1.94–2.14%) in octogenarians (390 strokes in 19 101 patients) versus 1.85% (95% CI, 1.75–1.95%) in nonoctogenarians (1395 strokes in 75 537 patients); *P*=0.046. Risk of perioperative MI was 1.39% (95% CI, 1.32–1.46%) overall, and 1.66% (1.55–1.77%) in octogenarians (141 MI in 8470 patients) versus 1.31% (95% CI, 1.24–1.38%) in nonoctogenarians (349 MI in 26 722 patients); *P*=0.009. Perioperative death was 0.65% (95% CI, 0.60–0.70%) overall, and 1.09% (95% CI, 0.94–1.25%) in octogenarians (203 deaths in 18 702 patients) versus 0.53% (95% CI, 0.48–0.59%) in nonoctogenarians (392 deaths in 73 327 patients); *P*<0.001. Meta-analysis of studies with age cut-points from 65 through 80 years revealed an increased risk of perioperative events across all age cut-points; maximal (OR, 1.28 [95% CI, 1.07–1.53]) for stroke, (OR, 1.83 [CI, 1.51-2.21]) for MI, and (OR, 2.37 [95% CI, 1.56–3.61]) for mortality (Figures S2 and S3). Sensitivity analysis in relation to study type revealed higher pooled perioperative stroke risk in elderly patients in registries (OR, 1.20 [95% CI, 1.07–1.35]), but not in trials (OR, 1.10 [95% CI, 0.95–1.26]), and cohort studies (OR, 1.06 [95% CI, 0.7–1.59]; Figure S4). When stratified by study quality, meta-analysis of high-quality studies showed consistent results with the main analysis (OR, 1.20 [95% CI, 1.06–1.35]). The age effect was not significant in meta-analysis of studies with low to medium quality (OR, 1.10 [95% CI, 0.97–1.26]; Figure S4).

**Figure 2.**
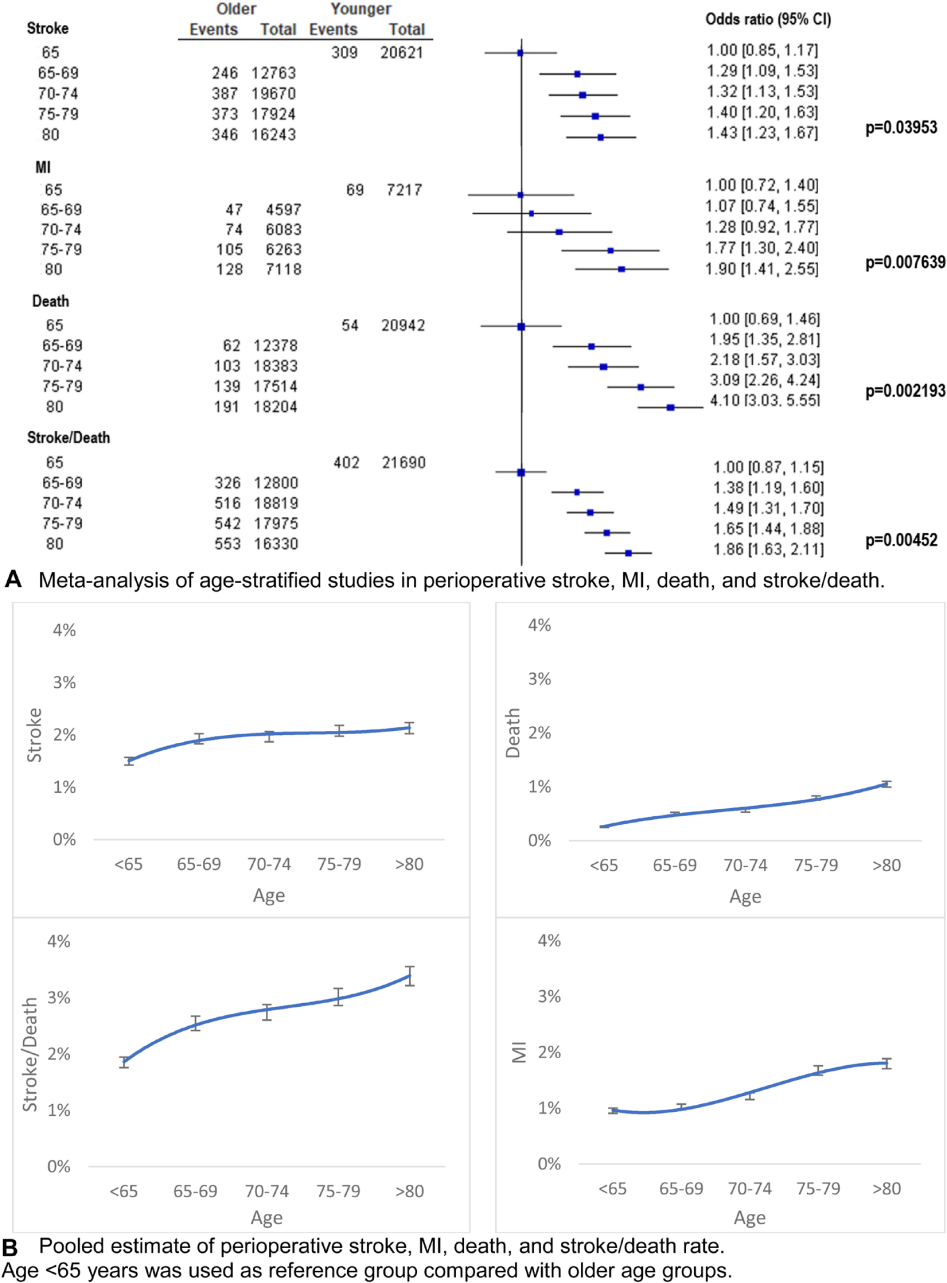
Meta-analysis and pooled estimate of perioperative stroke, myocardial infarction (MI), death, and stroke/death in age-stratified studies.

Six age-stratified studies with 87 614 patients (heterogeneity I^2^= 25%; *P*=0.25) revealed a linear association between 5-year increase in age (65–80 years) and perioperative stroke (linear trend *P*=0.040), MI (*P*=0.008), death (*P*=0.002), and stroke/death (*P*=0.005; Figure 2A). Every 5-year increase in age from 65 through 80 years was linearly associated with increased perioperative stroke by 0.142% (SE, 0.01; *P*=0.039), MI by 0.234% (SE, 0.02; *P*=0.0076), and death by 0.188% (SE, 0.01; *P*=0.0022; Figure 2B).

**Figure 3.**
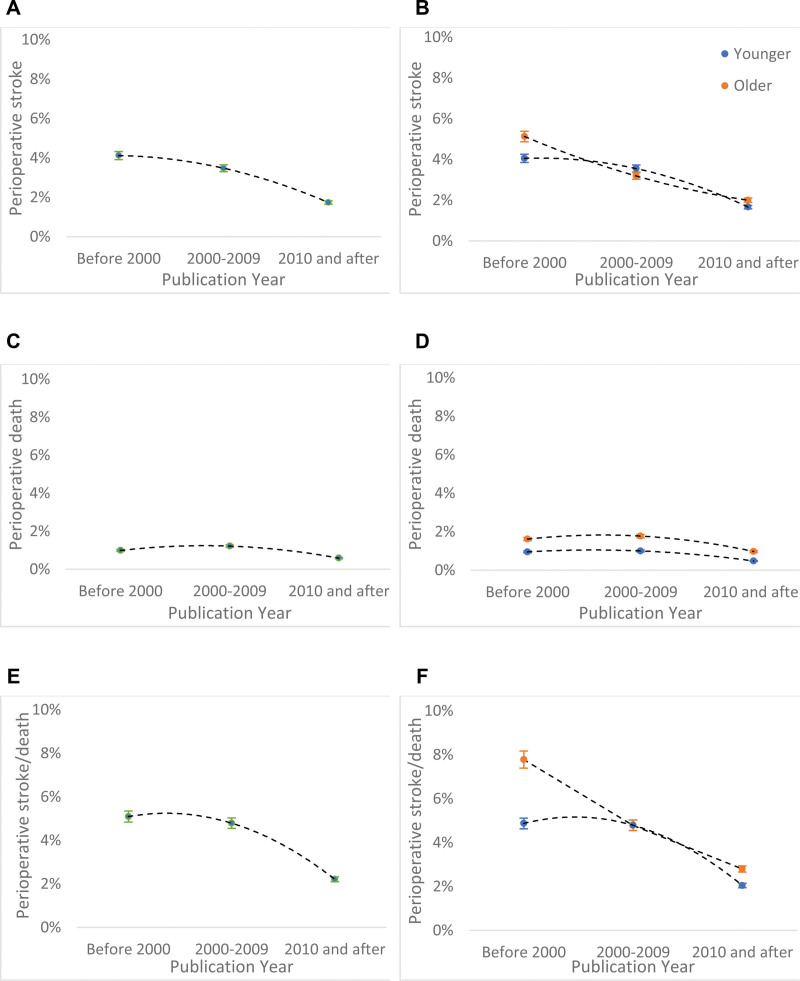
Time trend in pooled estimates of perioperative and annual of stroke and death rates in symptomatic carotid stenosis patients treated by carotid endarterectomy, stratified by age cut-off at 80 y.

In all studies, the pooled estimate of perioperative stroke risk declined across the 3 decades of analysis in all age groups, from 4.12% (95% CI, 4.04–4.26%) in studies published before 2000 through 1.74% (95% CI, 1.55–1.93%) after 2010 (Figure 3A and 3B). The decline was significant in both octogenarians (5.13% [95% CI, 3.13–7.92%] to 2.00% [95% CI, 1.80–2.21%]; *P*<0.001) and nonoctogenarians (4.06% [95% CI, 3.56–4.60%] to 1.66% [95% CI, 1.57–1.76%]; *P*<0.001). Perioperative death also declined over the 3 studied decades from 0.99% [95% CI, 0.72–1.32%] to 0.58% [95% CI, 0.54–0.63%]; *P*=0.001), in octogenarians (1.61% [95% CI, 1.56–1.66%] to 0.97% [95% CI, 0.26–1.65%]; *P*=0.04), and in nonoctogenarians (0.95% [95% CI, 0.69–1.29%] to 0.48% [95% CI, 0.43–0.53%]; *P*<0.001; Figure 3C and 3D). Perioperative stroke or death declined over the 3 decades in all age groups from 5.09% (95% CI, 4.98–5.20%) to 2.21% (95% CI, 2.05–2.37%), in octogenarians this declined from (7.78% [95% CI, 5.58–10.55%]) to (2.80% [95% CI, 2.56–3.04%]; *P*<0.001; Figure 3E and 3F).

Across all studies with outcomes beyond the perioperative period (n=14), median follow-up was 4.14 years (IQR 3–5 years; 186 616 patient-years). Annual stroke rate fell from 4.61% (95% CI, 3.89–5.42%) in studies published before 2005 to 3.46% (95% CI, 2.93–4.05%) after 2015 (*P*=0.006). Annual stroke rates in studies after 2015 were similar between octogenarians (3.65% [95% CI, 3.04–4.36%]) and nonoctogenarians (2.87% [95% CI, 1.96–4.04%]; *P*=0.109; Figure S5A and S5B). Annual mortality declined from 8.47% (95% CI, 6.94–10.23) to 1.01% (0.91–1.12; *P*<0.001). Annual death rates were higher in octogenarians than nonoctogenarians across the 3 studied decades. The rate difference reduced significantly from 1.72% (95% CI, 1.47–2.00%) in studies published before 2005 to 0.32% (95% CI, 0.22–0.45%) after 2015 (*P*<0.001; Figure S5C and S5D).

IPD from 4 prospective studies (n=4463) enabled multivariate analysis. Mean age was 71.8 years and 41.7% were female (Table S3, Figure S6). Multivariate risk factors analysis revealed age and index event type (stroke compared to transient ischemic attack or amaurosis fugax) were independently associated with perioperative stroke, MI, and death (Table). Age >85 years was independently associated with perioperative stroke (*P*=0.0021) and death (*P*=0.005). Pooled estimates of all studies (n=14; 186 616 patient-years follow-up) with results stratified by vascular risk factors showed higher annual stroke or death rates in octogenarians compared to nonoctogenarians when stratified by the prevalence of coronary artery disease: 1.92% (95% CI, 1.66–2.42%) versus 1.39% (95% CI, 1.17–1.64%) at <20% prevalence, *P*=0.002; and 7.3% (95% CI, 6.78–7.85%) versus 4.73% (95% CI, 4.13–5.18%) at >40% prevalence, *P*<0.001 (Figure S7).

**Table.**
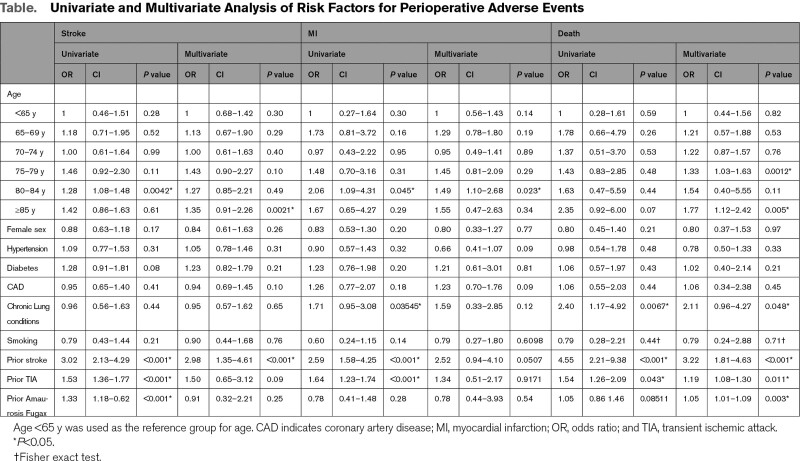
Univariate and Multivariate Analysis of Risk Factors for Perioperative Adverse Events

**Figure 4.**
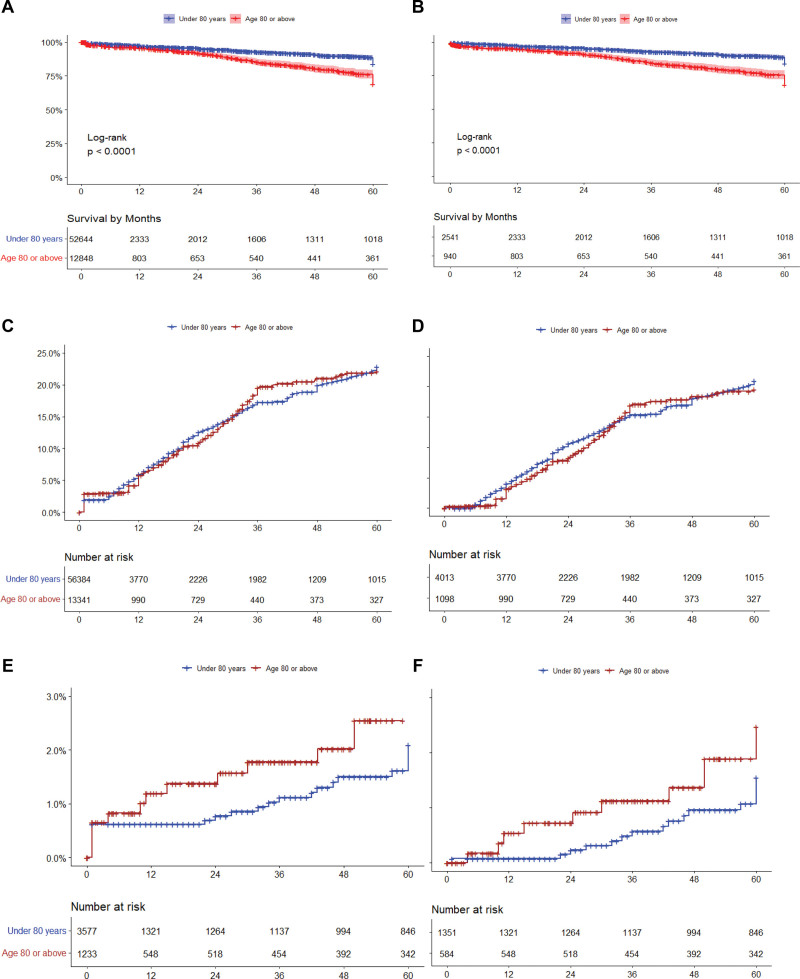
**Kaplan Meier curve of survival.** Kaplan Meier curve of survival including (**A**) and excluding (**B**) papers reporting perioperative events only, cumulative incidence of stroke including (**C**) and excluding (**D**) papers reporting perioperative events only, cumulative incidence of myocardial infarction including (**E**) and excluding (**F**) papers reporting perioperative events only.

Six studies provided Kaplan-Meier survival curves or crude stroke or death rates, which enabled extraction of patient-level data for survival analysis (n=65 492). Survival rates of 12 848 octogenarians and 52 644 nonoctogenarians were similar at 1-year (95.0% [95% CI, 93.2–96.5%] versus 97.5% [95% CI, 96.4–98.6%]; *P*=0.08) and 2-year postoperation (90.9% [95% CI, 88.3–93.5%] versus 95.7% [95% CI, 93.5–97.9%]; *P*=0.06). Five-year mortality was significantly higher in octogenarians (203 [21.6%; 95% CI, 16.6–26.6%] of the 940 patients) than the nonoctogenarians (268 [10.5%; 95% CI, 8.5–12.5%] of the 2541 patients; *P*<0.001; Figure 4A). Stroke rates were similar between octogenarians and nonoctogenarians at 1-year (4.86% [95% CI, 4.28–4.52%] versus 3.91% [95% CI, 2.99–4.11%]; *P*=0.07), 2-year (6.83% [95% CI, 5.37–8.56%] versus 8.65% [95% CI, 7.76–9.61%]; *P*=0.06), and 5-year postoperation (11.93% [95% CI, 9.98–14.16%] versus 12.78% [95% CI, 11.65–13.6%]; *P*=0.24; Figure 4C). Results were similar when studies reporting perioperative risks only were excluded (Figure 4B and 4D).

## Discussion

This is the first meta-analysis of safety of CEA by age for patients with symptomatic carotid stenosis, which includes all study types and individual patient-level data analysis. We have shown that age is linearly associated with risk of perioperative stroke, MI, and death; however, for elderly patients undergoing urgent CEA, risks have significantly declined over the last 3 decades. In IPD multivariate analysis, age and type of index cerebrovascular event were independently associated with risk of perioperative morbidity when adjusting for other key risk factors. Although symptom status is known to be strongly associated with recurrent stroke risk and post-CEA morbidity,^3,59^ this is the first study to confirm the association of age with both short- and long-term outcome in symptomatic patients.

Our analysis has revealed that the periprocedural period accounts of a majority of the postoperative adverse events, and the long-term risks of stroke and MI in octogenarians were similar to that of nonoctogenarians. Previous trials had confirmed that the risk of recurrent stroke increases significantly with age in recently symptomatic patients on best medical therapy alone.^9^ As our study found a modest increase in risk of perioperative morbidities in older patients, it would appear that selective early intervention in elderly symptomatic patients, particularly those without major comorbidity, will have an overall benefit in stroke risk reduction, comparable to that in younger patients. Although the 5-year survival was lower in octogenarians, the 1-year and 2-year survival did not differ, suggesting that the difference in long-term survival between older and younger patients is not related with the operative intervention.

Our findings have implications for clinical practice. First, the effect of age on perioperative morbidity was linear without a clear step-change in risk. This contrasts with the current notion of higher perioperative morbidity in the elderly once they reach a certain age. Although perioperative risk increased modestly with age, 1- and 2-year stroke risk were similar across age groups, suggesting that overall, the safety of CEA in older patients is acceptable. Second, the 1- and 2-year survival was comparable across age groups, which is compatible with the notion that the lower 5-year survival in octogenarians is unrelated to procedural risk and simply reflects life expectancy. Third, perioperative and annual stroke and death rates have declined over the last 3 decades, with steep declines found for older patients. This may be due to higher compliance rates with medical therapy in the elderly compared to younger patients, and safer anesthetic and procedural techniques in the current era. This data again supports appropriate intervention in elderly symptomatic patients who are fit for surgery. Fourth, cardiac risk is a major concern for elderly patients undergoing any intervention. We have confirmed that perioperative risk of MI increases modestly for older patients undergoing CEA. We also confirm that in pooled analysis of studies with a high prevalence of coronary artery disease, elderly patients have a higher annual risk of stroke and death post-CEA compared to younger patients. This key finding supports clinical caution in selecting elderly patients with significant coronary artery disease for CEA. Conversely, it also provides reassurance that for elderly patients without major cardiac comorbidity, outcome following CEA is similar to that of younger patients. In routine clinical practice, some elderly patients without significant comorbidity are excluded from surgery purely due to their age. This meta-analysis reveals that CEA is safe in symptomatic elderly patients who are selected for intervention, and therefore a key message is not to exclude symptomatic elderly patients from CEA just because of their age.

Our study is the first systematic review and meta-analysis of all study types focusing solely on symptomatic patients. Previous reviews of outcome by age have either focused largely on asymptomatic carotid disease or included only trial data. Investigating the safety of CEA in elderly asymptomatic patients is of more debatable clinical importance as intervention may confer less benefit due to limited life expectancy. Including only trial data in meta-analysis also has major limitations due to the few outcome events available in the elderly with limited follow-up, and few elderly patients being recruited into trials with age-related recruitment bias potentially undermining any age-risk association.^7^ Our focus purely on symptomatic carotid disease, with the inclusion of all published studies, enables detailed outcome analysis providing precise estimates of risk stratified by age, and guidance for contemporary clinical practice. Although this meta-analysis reveals the safety of selective surgery for elderly symptomatic patients without major comorbidities, there is a paucity of data on the effect of, and compliance with, contemporary best medical therapy in the elderly. Future clinical trials investigating the efficacy of surgery versus best medical therapy alone for very elderly symptomatic patients would be of clinical interest. However, eliminating selective recruitment bias in such trials would be very challenging and, therefore, interpreting results would require great caution.

Several limitations of this study should be noted. First, patients in studies published before 2010 may not be on contemporary intensive medical therapy for control of vascular risk factors. This may contribute to an overall higher risk of stroke and death than that found in current clinical practice. To account for this, our time-trend analyses have confirmed the changes in risk following surgery over the last 3 decades. Second, perioperative stroke as the study outcome was defined as all stroke, which included hemorrhagic stroke and contralateral stroke. Third, perioperative outcomes stratified by vascular risk factors were not available for all studies. Fourth, there are potential selection biases in which patients are offered intervention across all age groups in observational studies, randomized trials, and also routine clinical practice. Some patients, at any age, are turned down for intervention due to a spectrum of reasons including, adverse anatomy, delayed presentation, low-risk features such as borderline stenosis, high surgical risk, and patient choice. Although this meta-analysis cannot analyze the outcome of patients who are turned down for intervention and so not included in published studies, the results should be robust and representative of patients who are offered intervention in clinical practice. The inclusion of all identified published observational studies, rather than just trial data, is an important strength of this meta-analysis in this regard. In addition, we have performed sensitivity analyses of perioperative risk by study type. This revealed higher perioperative stroke risk in elderly patients included in registries compared to trials and cohort studies. Registries are generally agreed to be more inclusive and less selective than trials and so this analysis provides some insight on this subject area. Fifth, postoperative examination by a neurologist is not routine in some observational studies, which may contribute to under-detection of postoperative stroke in this meta-analysis. In sensitivity analysis, observational studies that were conducted with postoperative neurologist examination were graded as higher quality than those without. Analysis by study quality showed that the association of older age with higher perioperative stroke risk was preserved only in high-quality studies.

In conclusion, we found a linear increase in perioperative risk of stroke, MI, and death with age in symptomatic patients undergoing CEA. The 1- and 2-year mortality, and postoperative stroke rates up to 5 years postsurgery were comparable between older and younger patients. As stroke risk increases with age when on medical therapy alone, our findings support selective urgent intervention in symptomatic elderly patients, particularly those without major cardiac comorbidity.

## Article Information

### Acknowledgments

The work uses data provided by the National Vascular Registry. The National Vascular Registry is commissioned by the Healthcare Quality Improvement Partnership as part of the National Clinical Audit Program. The funders had no role in the study design, data collection or analysis, preparation of the article, or decision to publish the findings. The authors thank Mr Sam Waton for his support. The authors also thank Dr Eric Lim at the Department of Vascular, Endovascular and Transplant Surgery in Christchurch Hospital New Zealand for his support in providing subgroup data by age range. This study was partially supported by Chiang Mai University.

### Sources of Funding

This study was funded by the National Institute for Health Research (NIHR) Oxford Biomedical Research Center (BRC). The views expressed are those of the author(s) and not necessarily those of the National Health Service (NHS), the NIHR, or the Department of Health. The funders of the study had no role in study design, data collection, data analysis, data interpretation, or writing of the report.

### Disclosures

Dr Dardik declared that he is the Editor-in-Chief of the journal *JVS-Vascular Science* that is published by the Society for Vascular Surgery. The other authors report no conflicts.

### Supplemental Material

Figures S1–S7

Tables S1–S3

List S1

## Supplementary Material
