# Supplementary material for: Safety of Carotid Endarterectomy for Symptomatic Stenosis by Age: Meta-Analysis With Individual Patient Data
Source: Stroke. 2023 Jan 24;54(2):457–67. doi: 10.1161/STROKEAHA.122.040819 (PMC9855737; doi:10.1161/STROKEAHA.122.040819)

Supplementary materials legend

Table S1 Reporting checklist for meta-analysis of observational studies and for items that should be included in reports of cohort study

Figure S1 PRISMA diagram for systematic Review and Meta-analysis

List S1. Search terms

Table S2 Demographic details, study design, and surgical method used to perform carotid endarterectomy in all the publications.

Figure S2 Meta-analysis of rates of perioperative stroke (**Figure S2.1**), MI (**Figure S2.2**) and death (**Figure S2.3**) across age cut-offs.

Figure S3 Egger Funnel Plot for assessment of potential publication bias, including all studies published since 1980, reporting rates of perioperative stroke in patients with symptomatic carotid stenosis stratified by age groups

Figure S4 Sensitivity analysis of rates of perioperative stroke in patients with symptomatic carotid stenosis by age stratified by study type and grading of study quality

Figure S5 Time trend in pooled estimates of annual stroke and death rates, stratified by age cut-off at 80 years old

Table S3 Characteristics of the patients included in studies providing individual patient-level dataset

Figure S6 Egger Funnel Plot and Forest Plot for assessment of four prospective studies included in the individual patient-level data multivariate analysis

Figure S7 Annual stroke and death rate stratified by prevalence of vascular risk factors

Table S1 Reporting checklist for meta-analysis of observational studies and for items that should be included in reports of cohort study

Reporting checklist for meta-analysis of observational studies based on MOOSE reporting guidelines

		Reporting Item	Page Number
**Title**			
	#1	Identify the study as a meta-analysis of observational research	1
**Abstract**			
	#2	Provide a structured summary including, as applicable: background; objectives; data sources; study eligibility criteria, participants, and interventions; study appraisal and synthesis methods; results; limitations; conclusions and implications of key findings; systematic review registration number (From PRISMA checklist)	2
**Background**			
	#3a	Problem definition	2
	#3b	Hypothesis statement	4
	#3c	Description of study outcomes	2
	#3d	Type of exposure or intervention used	4
	#3e	Type of study designs used	4
	#3f	Study population	4
**Methods**			
Search strategy	#4a	Qualifications of searchers (eg, librarians and investigators)	4-5
Search strategy	#4b	Search strategy, including time period included in the synthesis and keywords	4-5, Supplementary material 2

Search strategy	#4c	Effort to include all available studies, including contact with authors	Supplementary material 3
Search strategy	#4d	Databases and registries searched	4
Search strategy	#4e	Search software used, name and version, including special features used (eg, explosion)	4, Supplementary material 2
Search strategy	#4f	Use of hand searching (eg, reference lists of obtained articles)	4, Supplementary material 3
Search strategy	#4g	List of citations located and those excluded, including justification	7, Supplementary material 3
Search strategy	#4h	Method of addressing articles published in languages other than English	4
Search strategy	#4i	Method of handling abstracts and unpublished studies	4
Search strategy	#4j	Description of any contact with authors	5
	#5a	Description of relevance or appropriateness of studies gathered for assessing the hypothesis to be tested	Supplementary material 5
	#5b	Rationale for the selection and coding of data (eg, sound clinical principles or convenience)	5
	#5c	Documentation of how data were classified and coded (eg, multiple raters, blinding, and interrater reliability)	5
	#5d	Assessment of confounding (eg, comparability of cases and controls in studies where appropriate)	6-7
	#5e	Assessment of study quality, including blinding of quality assessors; stratification or regression on possible predictors of study results	5-7
	#5f	Assessment of heterogeneity	6
	#5g	Description of statistical methods (eg, complete description of fixed or random effects models, justification of whether the chosen models account for predictors of study results, dose-response models, or cumulative meta-analysis) in sufficient detail to be replicated	5-6

	#5h	Provision of appropriate tables and graphics	Supplementary material 3,5
**Results**			
	#6a	Graphic summarizing individual study estimates and overall estimate	Figure 1-4
	#6b	Table giving descriptive information for each study included	Supplementary material 5
	#6c	Results of sensitivity testing (eg, subgroup analysis)	Supplementary material 7
	#6d	Indication of statistical uncertainty of findings	10
**Discussion**			
	#7a	Quantitative assessment of bias (eg. publication bias)	Supplementary material 11
	#7b	Justification for exclusion (eg, exclusion of non–English-language citations)	4
	#7c	Assessment of quality of included studies	Supplementary material 7
**Conclusion**			
	#8a	Consideration of alternative explanations for observed results	9-10
	#8b	Generalization of the conclusions (ie, appropriate for the data presented and within the domain of the literature review)	10
	#8c	Guidelines for future research	10
	#8d	Disclosure of funding source	10

Figure S1 PRISMA Diagram for Systematic Review and Meta-analysis

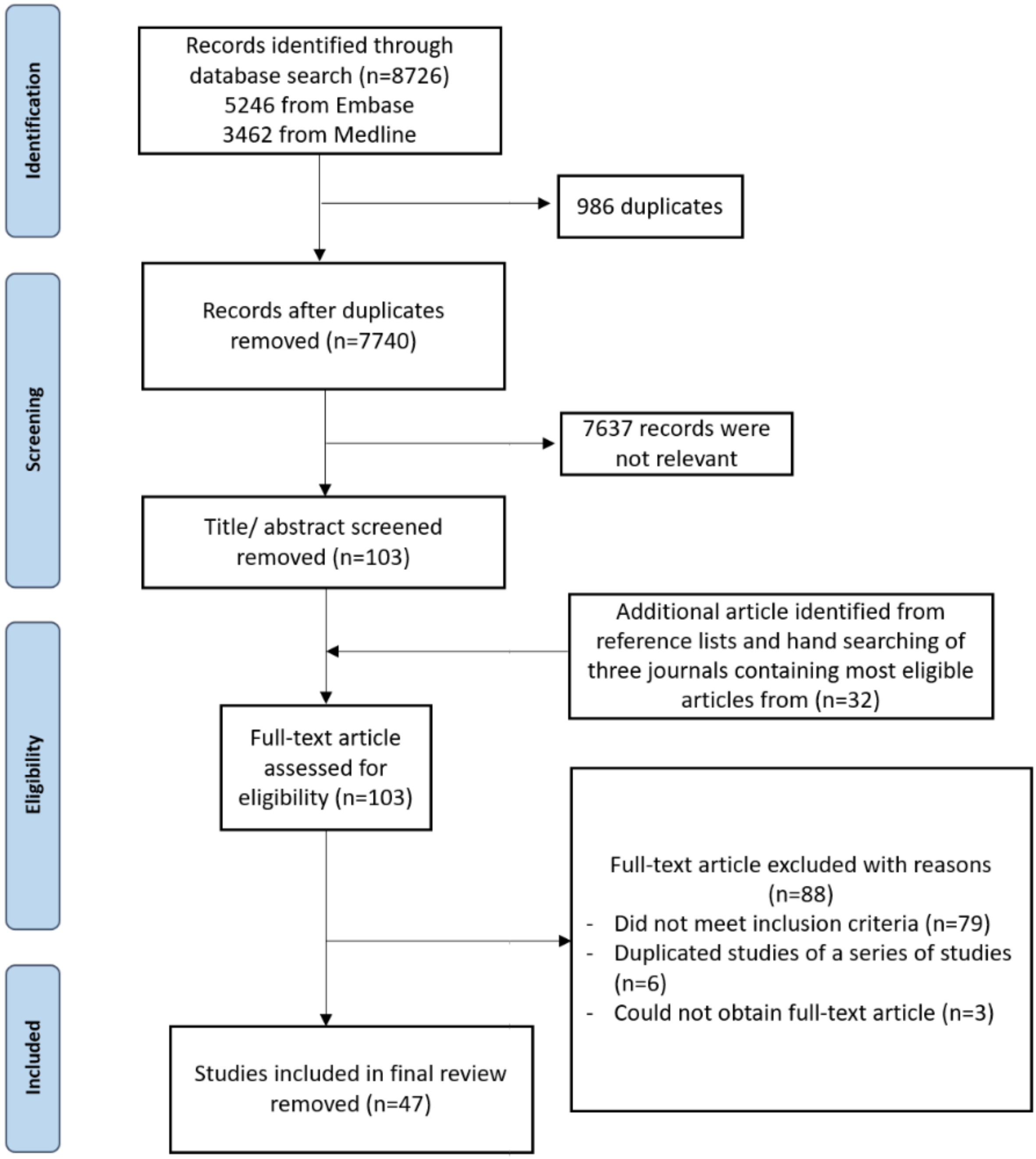

List S1 Search terms

MedLine (OVIDSp)

#1 exp embolic stroke/

#2 exp ischaemic stroke/

#3 exp thrombotic stroke/

#4 exp Ischemic Attack, Transient/

#5 #1 OR #2 OR #3 OR #4

#6 exp Carotid Artery Diseases/

#7 exp Carotid Stenosis/

#8 #6 OR #7

#9 #5 AND # 8

EMBASE (OVIDSp)

#1 Carotid Artery Disease OR Carotid Artery Stenosis OR Carotid Atherosclerosis

#2 Symptomatic OR TIA OR transient ischemic OR transient ischaemic OR stroke

#3 endarterectomy OR stent OR stenting OR surgery

#1 AND #2 AND #3

PubMed (ePub ahead of print only)

#1 Carotid Artery Disease OR Carotid Artery Stenosis OR Carotid Atherosclerosis

#2 Symptomatic OR TIA OR transient ischemic OR transient ischaemic OR stroke

#3 endarterectomy OR stent OR stenting OR surgery

#4 #1 AND #2 AND #3

#5 random* OR blind* OR "control group" OR placebo* OR controlled OR groups OR trial* OR "systematic review" OR "meta-analysis" OR metaanalysis OR "literature search" OR medline OR pubmed OR cochrane OR) AND (publisher[sb] OR inprocess[sb] OR pubmednotmedline[sb])

#6 #4 AND #5

(((Carotid Artery Disease OR Carotid Artery Stenosis OR Carotid Atherosclerosis) AND (Symptomatic OR TIA OR transient ischemic OR transient ischaemic OR stroke)) AND (endarterectomy OR stent OR stenting)) AND (random* OR blind* OR "control group" OR placebo* OR controlled OR groups OR trial* OR "systematic review" OR "meta-analysis" OR metaanalysis OR "literature search" OR medline OR pubmed OR cochrane OR cohort) AND (publisher[sb] OR inprocess[sb] OR pubmednotmedline[sb]))

Web of Science Conference Proceedings Citation Index (CPCI-S)

#1 TS=(Carotid Artery Disease OR Carotid Artery Stenosis OR Carotid Artery Narrowing)

#2 TS=(Endarterectomy OR stent OR stenting)

#3 TS=(symptomatic OR TIA OR stroke OR transient ischaemic OR transient ischemic)

#4 TS=(randomized OR randomised OR randomly OR controlled trial OR controlled study OR control group OR control groups OR double blind OR blind study OR blind trial)

#5 TS=(observational OR observation OR cohort)

#6 #5 AND #3 AND #2 AND #1

#7 #4 AND #3 AND #2 AND #1

#8 #6 OR #7

CENTRAL

ID Search

#1 MeSH descriptor: [Carotid Artery Disease] explode all trees

#2 MeSH descriptor: [Carotid Stensosi] explode all trees

#3 #1 or #2

#4 endarterectomy

#5 stent

#6 #4 OR #5

#7 #3 AND #6

#8 MeSH descriptor: [Ischaemic Attack, transient] explode all trees

#9 MeSH descriptor: [Stroke, Acute] explode all trees

#10 #8 OR #9

#11 #7 AND #10

1980 to 2021

Clinicaltrials.gov

Search terms: Carotid Artery Disease OR Carotid Occlusion OR Carotid Stenosis OR Carotid Artery Plaque OR Carotid Atherosclerosis

Type of Studies: all

Intervention: Endarterectomy OR stent OR stenting

Study Results: Studies with Results

WHO International Clinical Trials Registry

Condition: Carotid artery disease OR carotid occlusion OR carotid stenosis OR carotid artery plaque OR carotid atherosclerosis

Intervention: Endarterectomy OR stent OR stenting

Recruitment: ALL

Table S2 Demographic details, study design, and surgical method used to perform CEA in all the publications.

Ref no.	Study	Start Recruit	Finish Recruit	Median Recruit	Publication	Type of study	Mean Age	Male	Number of Patients included in analysis	Perioperative Stroke Rate	Diabetes	Hyper-lipidaemia	CAD	Region	Shunting	General Anaesthesia
1	ECST	1981	1994	1987	1998	Trial	60	70%	1745	0.067	12%	6%*	NG	USA	NG	NG
2	NASCET	1988	1996	1992	2001	Trial	NG	69.50%	1415	0.06	13.20%	26.40%	45%	western Europe, Canada	40%	95%
3	ACSNSQIP	1990	2002	1996	2015	Registry	70.53	59%	2459	0.046	25.99%	NG	17.81%	USA	NG	NG
4	CAVATAS	1992	1997	1994	2009	Trial	68	70%	228	0.087	13%	32%	39.90%	United States and Canada	64%	93%
5	CAPOCCIA	2005	2009	2007	2011	Prospective cohort	68	82.20%	62	0	33.80%	30.60%	32.20%	Italy	21%	100%
6	D'ORIA	2015	2019	2017	2022	Retrospective cohort	74.58	66.40%	157	0.019	38.90%	NG	23.50%	Italy	NG	100%
7	DANDAPANI	1994	1995	1996	1999	Retrospective cohort	70.8	55.0%	175	0.037	25.71%	62.28%	46.86%	USA	100%	100%
8	DE RANGO	2001	2010	2005	2012	Retrospective cohort	82.4	76.80%	99	0.062	17.70%	27.40%	29.00%	Italy	NG	NG
9	DEWEESE	1961	1966	1963	1973	Retrospective cohort	NG	63.11%	103	0.06	18%	77.19%	24%	USA	1.94%	91.26%
10	NIS (GILES)	2004	2007	2005	2010	Registry	71.1	57.50%	5,499	0.059†	NG	NG	10.80%	USA	NG	NG
11	GOLDSTEIN	1988	1990	1989	1994	Retrospective cohort	NG	63.30%	697	0.057	NG	NG	NG	USA	NG	NG
12	HALLETT	1970	1995	1982	1998	Retrospective cohort	67	65%	254	0.039	21%	NG	43%	USA	NG	100%
13	SWEDVASC	2008	2017	2012	2022	Registry	72	67%	7349	0.036	19.90%	NG	29%	Sweden	NG	NG
14	HOBBS	1994	2017	2005	2020	Retrospective cohort	92	47.72%	44	0.023	13.60%	45.50%	43.20%	USA	2.23%	4.55%

Ref no.	Study	Start Recruit	Finish Recruit	Median Recruit	Publication	Type of study	Mean Age	Male	Number of Patients	Perioperative Stroke Rate	Diabetes	Hyper-lipidaemia	CAD	Region	Shunting	General Anaesthesia
15	HOWARD	2000	2015	2008	2016	Meta-analysis of trials	NG	69.77%	4754	0.029	25.14%	68.51%	29.65%	Central and Western Europe, USA	NG	NG
16	HUANG	2003	2012	2007	2018	Retrospective cohort	72	68%	233	0.021	16%	78%	43%	USA	21%	100%
17	ICSS	2000	2010	2005	2010	Trial	70	70.40%	821	0.028	21%	66%	18%	UK	39.50%	20.80%
18	KARPENKO	2009	2017	2013	2020	Retrospective cohort	64.5	77.70%	364	0.025	23.60%	41.60%	91.40%	Russia	5.20%	100%
19	KASTRUP	1997	2004	2000	2004	Prospective cohort	78	64%	69	0.029	30%	31%	21%	Germany	90%	100%
20	LAU	1995	2004	1999	2005	Retrospective cohort	69.93	100%	286	0.014	33.47%	NG	42.68%	USA	NG	100%
21	LIM	2014	2017	2015	2020	Retrospective cohort	76	63.40%	172	0	22.09%	33.72%	41.86%	New Zealand	NG	NG
22	LOFTUS	1978	1986	1882	1988	Retrospective cohort	73.4	71.70%	53	0.0192	13%	NG	43%	USA	11%	100%
23	MAXWELL	1979	1988	1983	2000	Retrospective cohort	71.8	54.40%	1,608	0.019	16.04%	NG	29.63%	USA	33%	22.70%
24	MEYER	1971	1989	1980	1991	Retrospective cohort	78	NG	693	0.031	NG	NG	NG	USA	NG	NG
25	JVR	1993	2004	1998	2005	Registry	NG	59.25	842	0.011	75.10%	NA	52%	USA	100%	100%
26	NUNN	1963	1986	1976	1988	Retrospective cohort	66	62.50%	556	0.024	16%	15.20%	47.10%	USA	100%	100%
27	POL	2005	2010	2007	2013	Prospective cohort	69	72.40%	495	0.03	22%	67%	40%	Netherland	8%	100%
28	PRUNER	1995	2000	1997	2003	Prospective cohort	68.4	55.10%	2847	0.015	20.10%	45.60%	35.00%	Italy	20.30%	11%
29	CARE	2005	2011	2008	2013	Registry	78.1	59%	1376	0.028	32.40%	79.60%	NG	USA	NG	NG
30	REED	1990	1999	1994	2003	Retrospective cohort	69	57%	626	0.016	22%	NG	NG	USA	NG	NG
31	RINCKENBACH	1991	2003	1997	2007	Retrospective cohort	83.5	652.80%	24	0	24.30%	18.60%	15.70%	France	1.20%	95%

Ref no.	Study	Start Recruit	Finish Recruit	Median Recruit	Publication	Type of study	Mean Age	Male	Number of Patients	Perioperative Stroke Rate	Diabetes	Hyper-lipidaemia	CAD	Region	Shunting	General Anaesthesia
32	SPACE	2001	2006	2003	2006	Trial	68·2	71.60%	56,336	0.06	28.40%	NG	24%	Germany, Austria, and Switzerland	NG	NG
33	SALOMON DU MONT	2002	2007	2004	2014	Retrospective cohort	83.3	59.30%	50	0.02*	26.10%	54.60%	31.90%	France	12.90%	2%
34	GNSQAD	2009	2014	2011	2017	Registry	70.7	67.80%	56,336	0.02	NG	NG	NG	Germany	43%	71.30%
35	SHARPE	2008	2013	2010	2013	Retrospective cohort	NG	NG	475	0.013*	22%	NA	26%	UK	100%	100%
36	CREST	2000	2008	2004	2011	Trial	68.8	65.40%	1321	0.06*	27.50%	81.10%	39.30%	USA	NG	NG
37	CHACDP	2012	2012	2012	2005	Registry	NG	63.00%	1609	0.046*	28.70%	NG	NG	USA	33.30%	87.40%
38	TING	1993	1998	1995	2000	Retrospective cohort	82	67.34%	49	0.051	16%	NG	67%	USA	100%	100%
39	TREIMAN	1964	1990	1977	1992	Retrospective cohort	83	NG	110	0.02	NG	NG	NG	USA	96.30%	100%
40	VARGHESE	1990	2001	2005	2004	Retrospective cohort	69.9	69%	329	0.0529	NG	NG	NG	Australia	100%	100%
41	WADA	2005	2013	2009	2014	Retrospective cohort	73.3	86.67%	90	0.033‡	25.20%	20.10%	16.30%	Japan	NG	NG
42	NVR	2008	2021	2014	2022	Registry	NG	67.12%	28,048	0.012	23.27%	NG	29.65%	UK	54.71	86.71%
43	RECAS	2013	2016	2014	2021	Prospective cohort	64.2	85.90%	1176	0.041	26.30%	19.90%	15.10%	China	NG	96.80%
44	YANG	2012	2016	2014	2018	Retrospective cohort	63.3	77.78%	108	0.0185	28.70%	27.80%	14.80%	China	59.30%	100%
45	ZBORNIKOVA	1982	1987	1084	1998	Prospective cohort	63	25.00%	64	0.17	9%	NG	22%	Sweden	72%	100%
46	ACSNSQIP (ZHOLANJI)	2011	2015	2013	2018	Registry	NG	NG	NG	0.049	NG	NG	NG	USA	NG	NG

*lipid-lowering drugs

†stroke or death rate

‡ stroke or MI or death rate

Figure S2 Meta-analysis of rates of perioperative stroke (**Figure S2.1**), MI (**Figure S2.2**) and death (**Figure S2.3**) across age cut-offs.

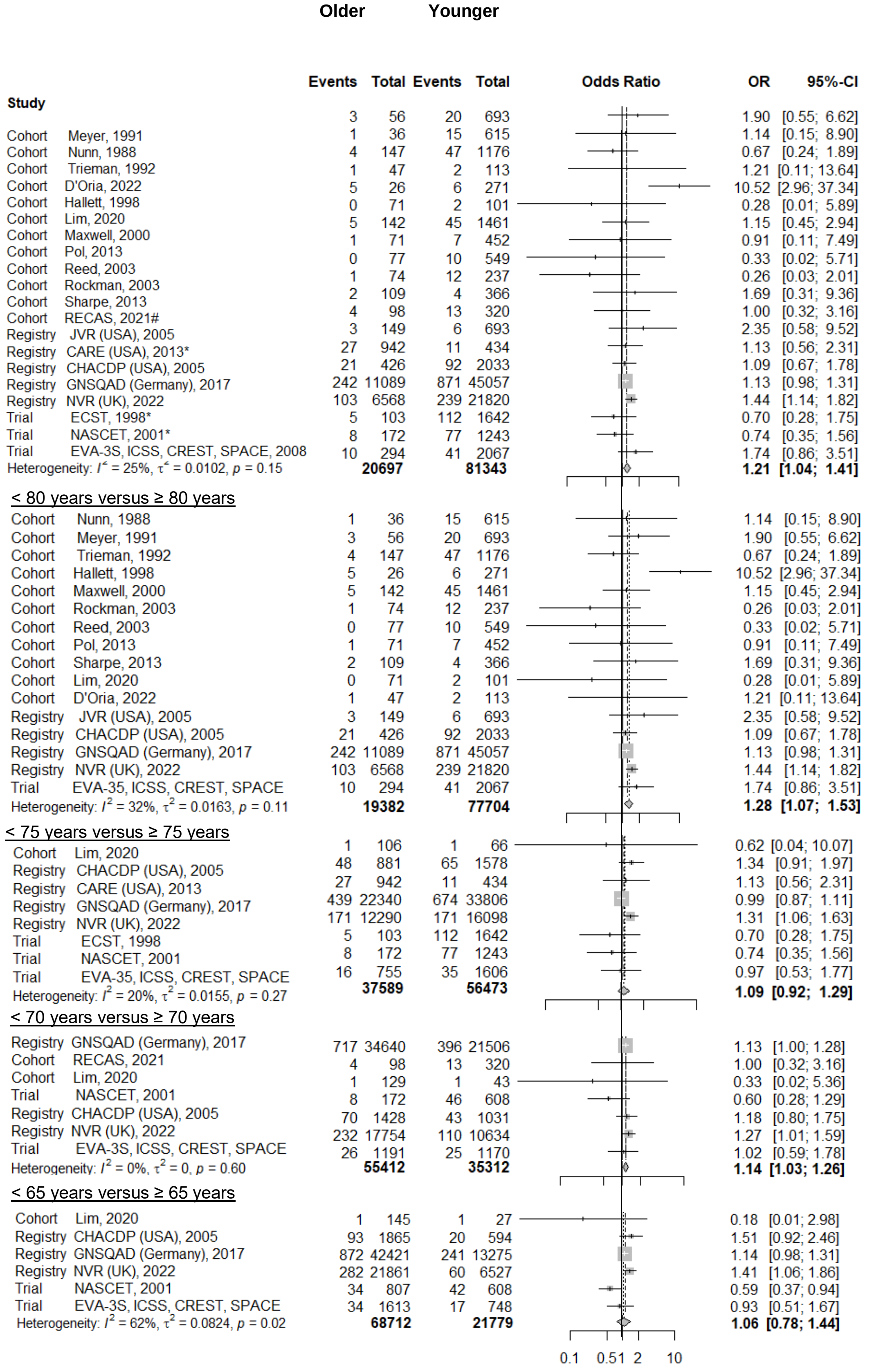

Figure S2.1 Meta-analysis of perioperative stroke by age cut-offs. Odds ratios (boxes/diamonds) greater than 1 indicate higher risk in older versus younger patients

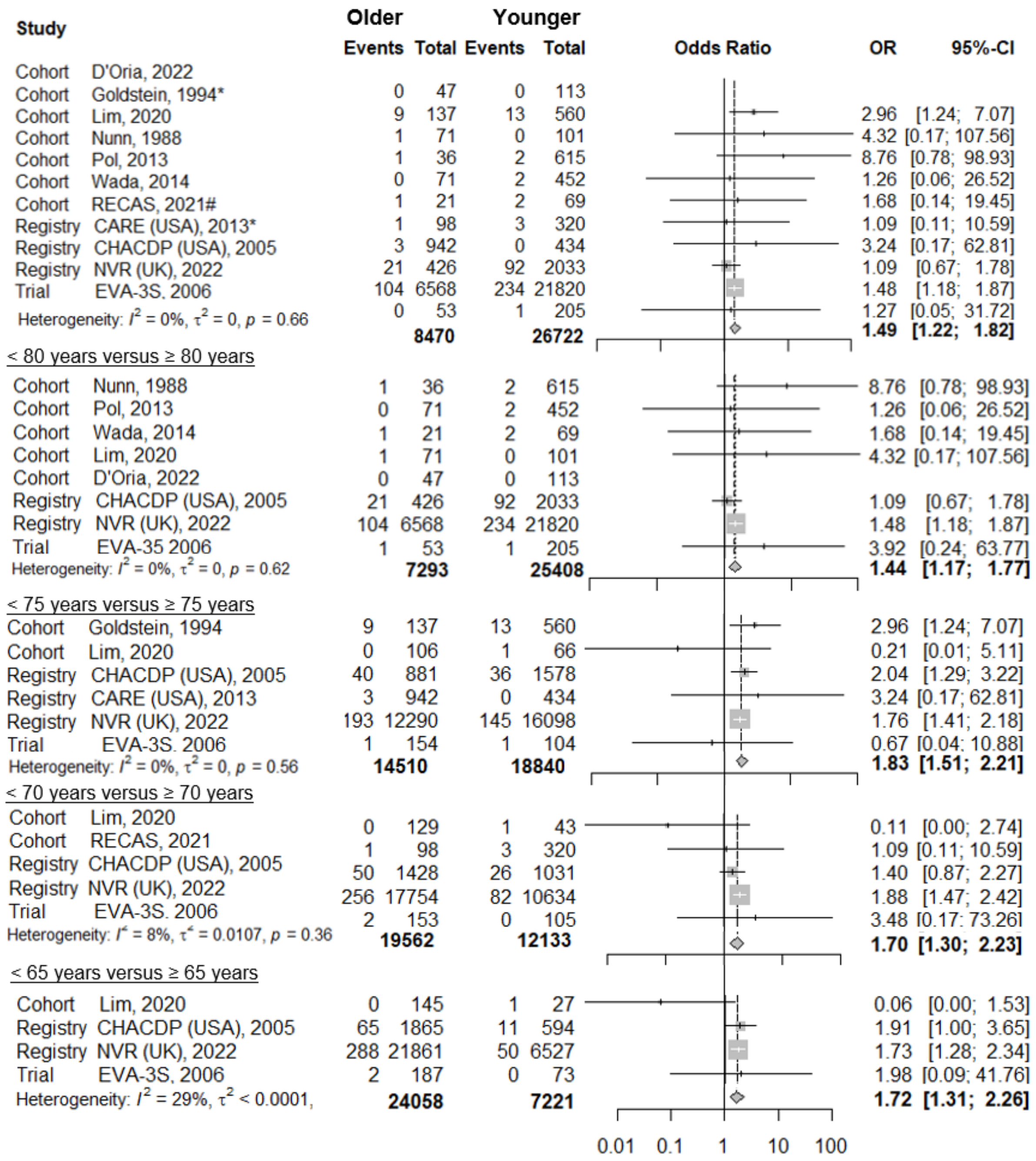

Figure S2.2 Meta-analysis of perioperative MI by age cut-offs. Odds ratios (boxes/diamonds) greater than 1 indicate higher risk in older versus younger patients

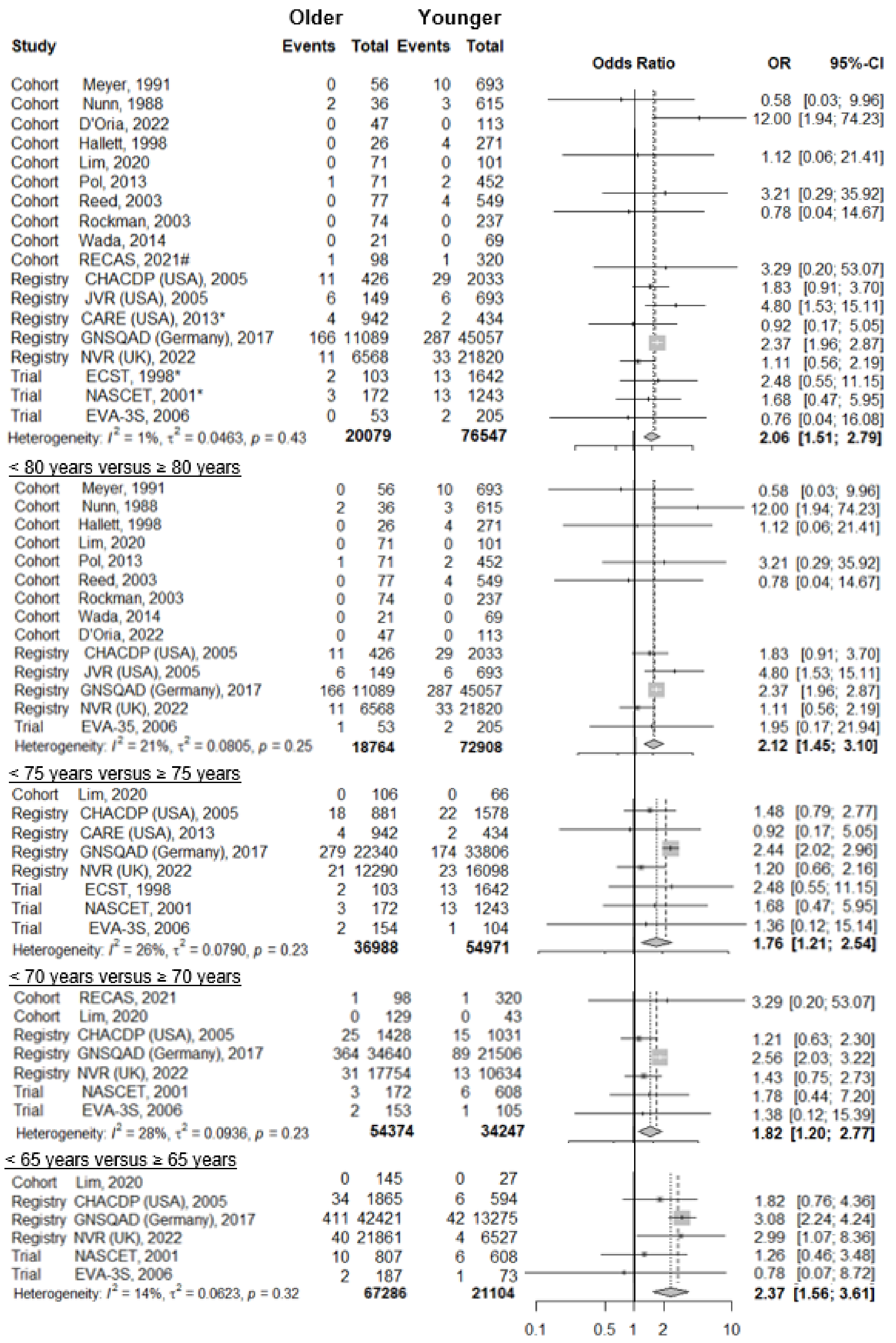

Figure S2.3 Meta-analysis of perioperative death by age cut-offs. Odds ratios (boxes/diamonds) greater than 1 indicate higher risk in older versus younger patients

Figure S3 Egger Funnel Plot for assessment of potential publication bias, including all studies published since 1980, reporting rates of perioperative stroke in patients with symptomatic carotid stenosis stratified by age

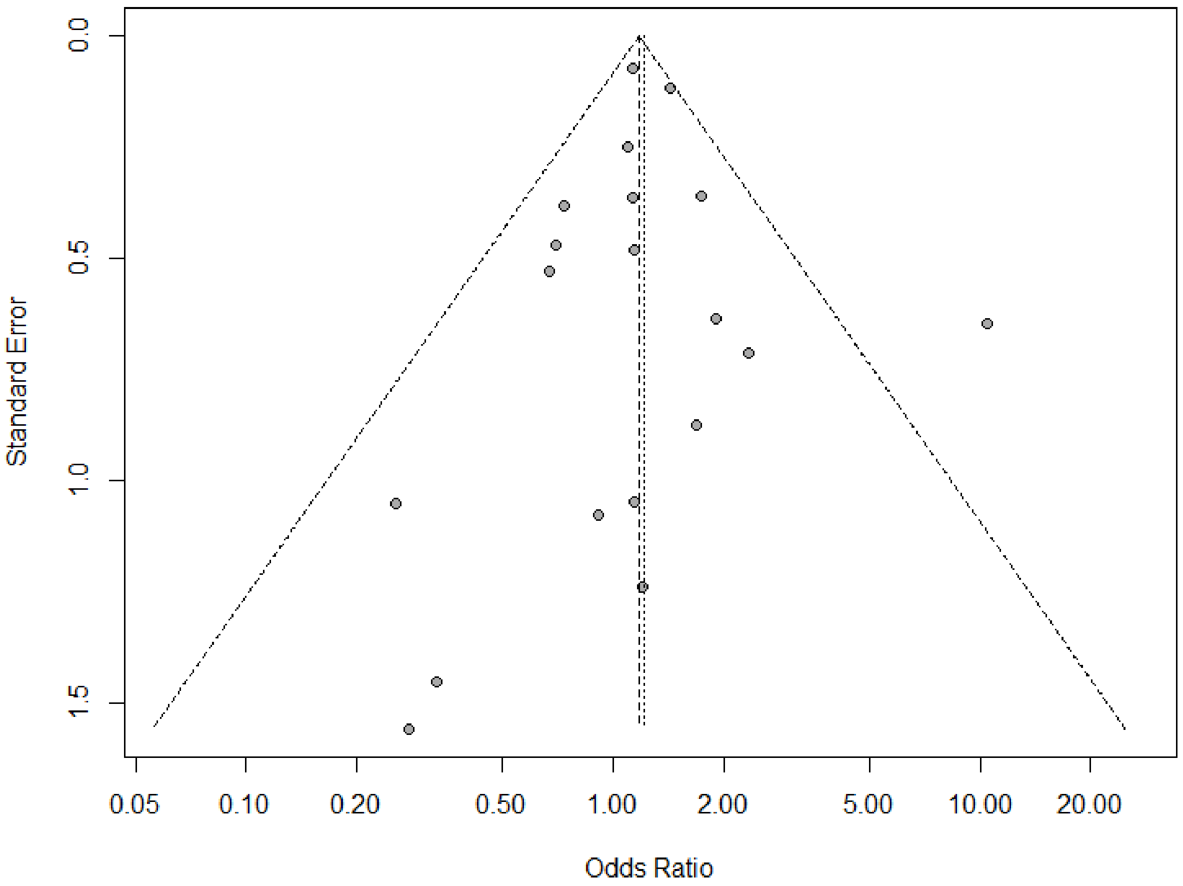

Figure S4 Sensitivity analysis of rates of perioperative stroke in patients with symptomatic carotid stenosis by age stratified by study type and grading of study quality

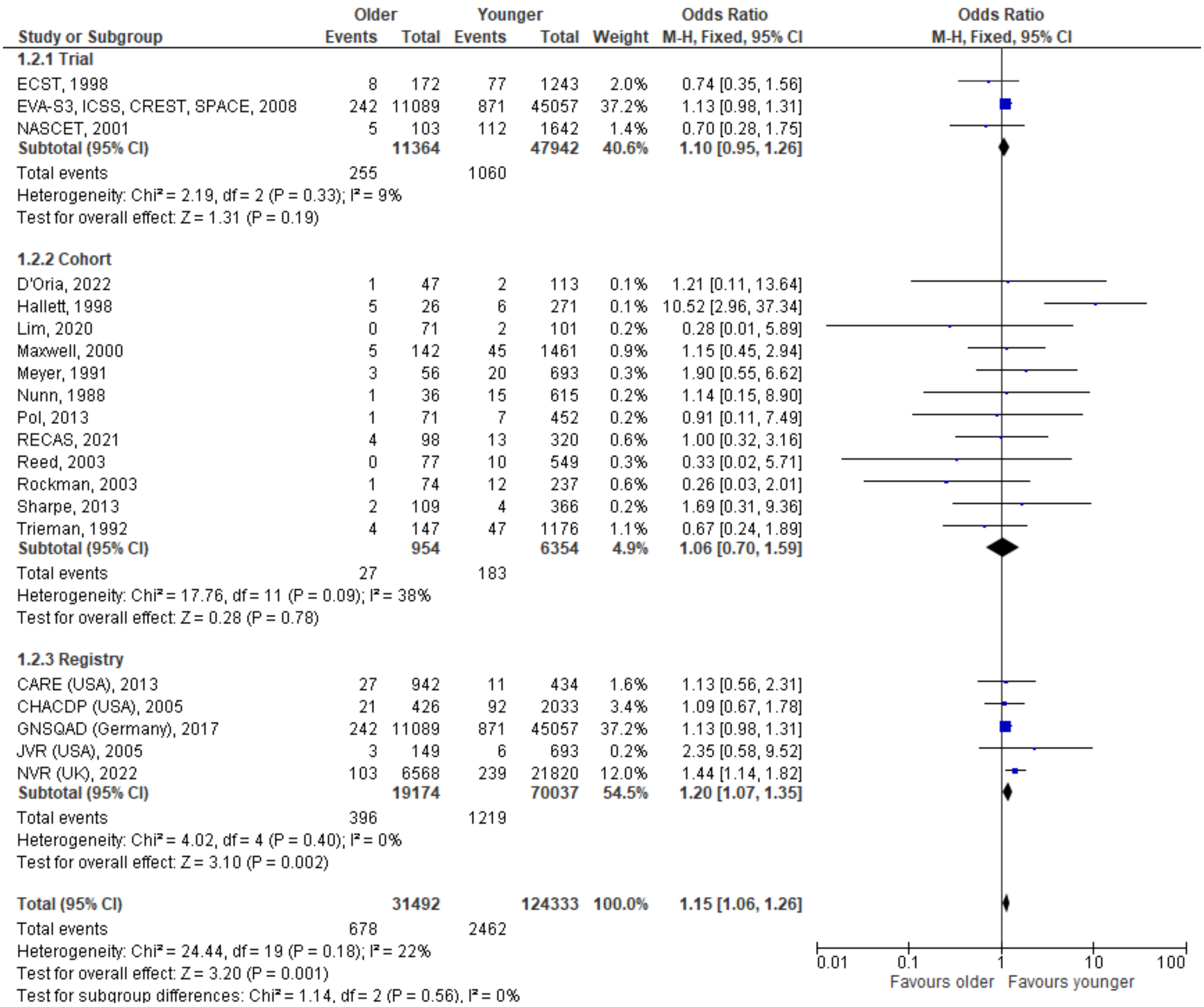

Study or Subgroup	Older Events	Older Total	Younger Events	Younger Total	Weight	Odds Ratio M-H, Fixed, 95% CI
**1.2.1 Trial**						
ECST, 1998	8	172	77	1243	2.0%	0.74 [0.35, 1.56]
EVA-S3, ICSS, CREST, SPACE, 2008	242	11089	871	45057	37.2%	1.13 [0.98, 1.31]
NASCET, 2001	5	103	112	1642	1.4%	0.70 [0.28, 1.75]
**Subtotal (95% CI)**		**11364**		**47942**	**40.6%**	**1.10 [0.95, 1.26]**
Total events	255		1060			
Heterogeneity: Chi² = 2.19, df = 2 (P = 0.33); I² = 9%						
Test for overall effect: Z = 1.31 (P = 0.19)						
**1.2.2 Cohort**						
D'Oria, 2022	1	47	2	113	0.1%	1.21 [0.11, 13.64]
Hallett, 1998	5	26	6	271	0.1%	10.52 [2.96, 37.34]
Lim, 2020	0	71	2	101	0.2%	0.28 [0.01, 5.89]
Maxwell, 2000	5	142	45	1461	0.9%	1.15 [0.45, 2.94]
Meyer, 1991	3	56	20	693	0.3%	1.90 [0.55, 6.62]
Nunn, 1988	1	36	15	615	0.2%	1.14 [0.15, 8.90]
Pol, 2013	1	71	7	452	0.2%	0.91 [0.11, 7.49]
RECAS, 2021	4	98	13	320	0.6%	1.00 [0.32, 3.16]
Reed, 2003	0	77	10	549	0.3%	0.33 [0.02, 5.71]
Rockman, 2003	1	74	12	237	0.6%	0.26 [0.03, 2.01]
Sharpe, 2013	2	109	4	366	0.2%	1.69 [0.31, 9.36]
Trieman, 1992	4	147	47	1176	1.1%	0.67 [0.24, 1.89]
**Subtotal (95% CI)**		**954**		**6354**	**4.9%**	**1.06 [0.70, 1.59]**
Total events	27		183			
Heterogeneity: Chi² = 17.76, df = 11 (P = 0.09); I² = 38%						
Test for overall effect: Z = 0.28 (P = 0.78)						
**1.2.3 Registry**						
CARE (USA), 2013	27	942	11	434	1.6%	1.13 [0.56, 2.31]
CHACDP (USA), 2005	21	426	92	2033	3.4%	1.09 [0.67, 1.78]
GNSQAD (Germany), 2017	242	11089	871	45057	37.2%	1.13 [0.98, 1.31]
JVR (USA), 2005	3	149	6	693	0.2%	2.35 [0.58, 9.52]
NVR (UK), 2022	103	6568	239	21820	12.0%	1.44 [1.14, 1.82]
**Subtotal (95% CI)**		**19174**		**70037**	**54.5%**	**1.20 [1.07, 1.35]**
Total events	396		1219			
Heterogeneity: Chi² = 4.02, df = 4 (P = 0.40); I² = 0%						
Test for overall effect: Z = 3.10 (P = 0.002)						
**Total (95% CI)**		**31492**		**124333**	**100.0%**	**1.15 [1.06, 1.26]**
Total events	678		2462			
Heterogeneity: Chi² = 24.44, df = 19 (P = 0.18); I² = 22%						
Test for overall effect: Z = 3.20 (P = 0.001)						
Test for subgroup differences: Chi² = 1.14, df = 2 (P = 0.56), I² = 0%						

Figure S4.1 Sensitivity analysis of rates of perioperative stroke in patients with symptomatic carotid stenosis by age stratified by study type. Odds ratios (boxes/diamonds) greater than 1 indicate higher risk in older versus younger patients

Study or Subgroup	Older Events	Older Total	Younger Events	Younger Total	Weight	Odds Ratio M-H, Fixed, 95% CI
**1.1.1 High quality**						
Reed, 2003	0	77	10	549	0.3%	0.33 [0.02, 5.71]
RECAS, 2021	4	98	13	320	0.6%	1.00 [0.32, 3.16]
NVR (UK), 2022	103	6568	239	21820	12.0%	1.44 [1.14, 1.82]
Maxwell, 2000	5	142	45	1461	0.9%	1.15 [0.45, 2.94]
EVA-S3, ICSS, CREST, SPACE, 2008	242	11089	871	45057	37.2%	1.13 [0.98, 1.31]
D'Oria, 2022	1	47	2	113	0.1%	1.21 [0.11, 13.64]
CARE (USA), 2013	27	942	11	434	1.6%	1.13 [0.56, 2.31]
**Subtotal (95% CI)**		**18963**		**69754**	**52.8%**	**1.20 [1.06, 1.35]**
Total events	382		1191			
Heterogeneity: Chi² = 3.88, df = 6 (P = 0.69); I² = 0%						
Test for overall effect: Z = 2.96 (P = 0.003)						
**1.1.2 Low and medium quality**						
Trieman, 1992	4	147	47	1176	1.1%	0.67 [0.24, 1.89]
Sharpe, 2013	2	109	4	366	0.2%	1.69 [0.31, 9.36]
Rockman, 2003	1	74	12	237	0.6%	0.26 [0.03, 2.01]
Pol, 2013	1	71	7	452	0.2%	0.91 [0.11, 7.49]
Nunn, 1988	1	36	15	615	0.2%	1.14 [0.15, 8.90]
NASCET, 2001	5	103	112	1642	1.4%	0.70 [0.28, 1.75]
Meyer, 1991	3	56	20	693	0.3%	1.90 [0.55, 6.62]
Lim, 2020	0	71	2	101	0.2%	0.28 [0.01, 5.89]
JVR (USA), 2005	3	149	6	693	0.2%	2.35 [0.58, 9.52]
Hallett, 1998	5	26	6	271	0.1%	10.52 [2.96, 37.34]
GNSQAD (Germany), 2017	242	11089	871	45057	37.2%	1.13 [0.98, 1.31]
ECST, 1998	8	172	77	1243	2.0%	0.74 [0.35, 1.56]
CHACDP (USA), 2005	21	426	92	2033	3.4%	1.09 [0.67, 1.78]
**Subtotal (95% CI)**		**12529**		**54579**	**47.2%**	**1.10 [0.97, 1.26]**
Total events	296		1271			
Heterogeneity: Chi² = 20.07, df = 12 (P = 0.07); I² = 40%						
Test for overall effect: Z = 1.51 (P = 0.13)						
**Total (95% CI)**		**31492**		**124333**	**100.0%**	**1.15 [1.06, 1.26]**
Total events	678		2462			
Heterogeneity: Chi² = 24.44, df = 19 (P = 0.18); I² = 22%						
Test for overall effect: Z = 3.20 (P = 0.001)						
Test for subgroup differences: Chi² = 0.80, df = 1 (P = 0.37), I² = 0%						

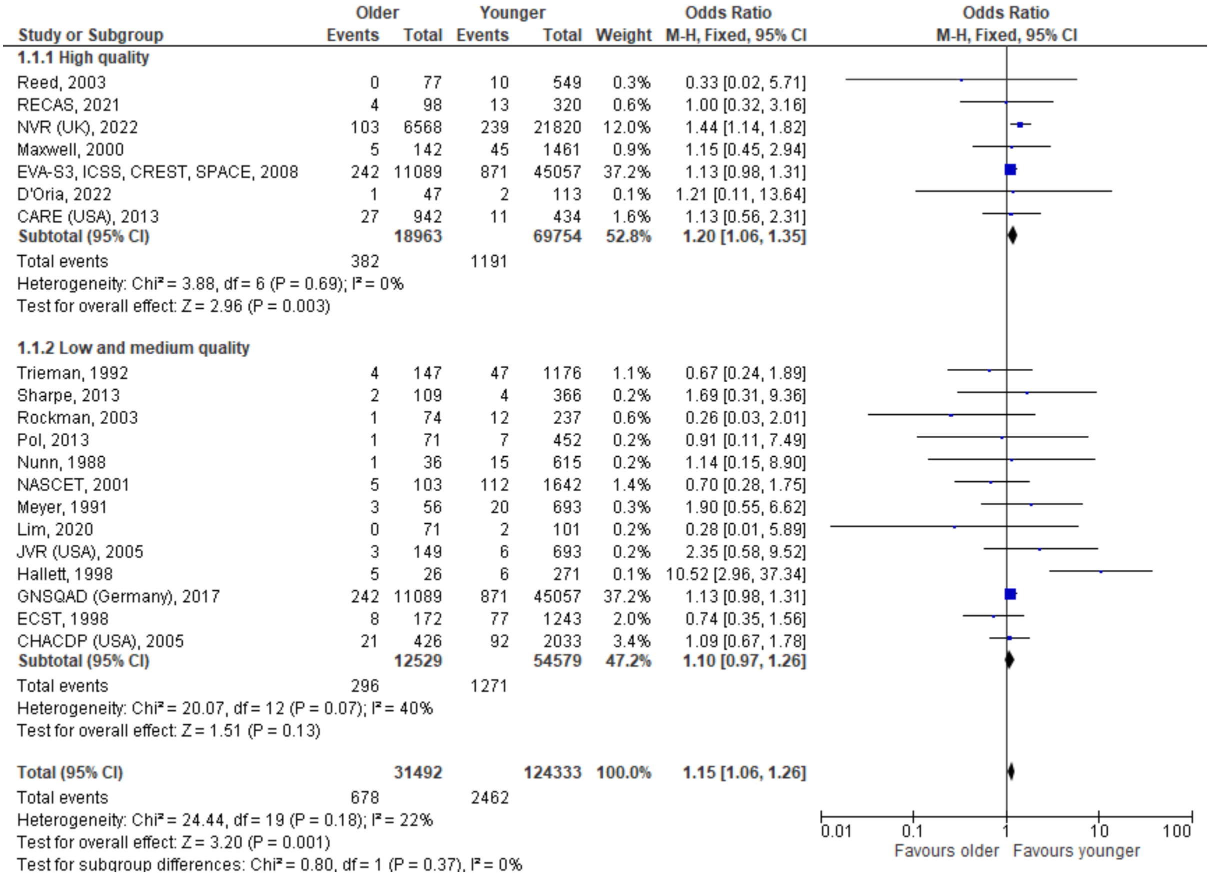

Figure S4.2 Sensitivity analysis of rates of perioperative stroke in patients with symptomatic carotid stenosis by age stratified by grading of study quality and study type – high versus low-to-medium. Odds ratios (boxes/diamonds) greater than 1 indicate higher risk in older versus younger patients

Study quality graded based on STROBE criteria and key criteria required for meta-analysis, including the following items: study objectives stated clearly; patient selection criteria stated clearly; patients enrolled consecutively without predetermined selection; interventions adequately described; outcome definitions provided; rate of dropout of <20%, and outcome ascertainment by a neurologist.

Figure S5 Time trend in pooled estimates of annual stroke and death rates in symptomatic carotid stenosis patients treated by carotid endarterectomy, stratified by age cut-off at 80 years old

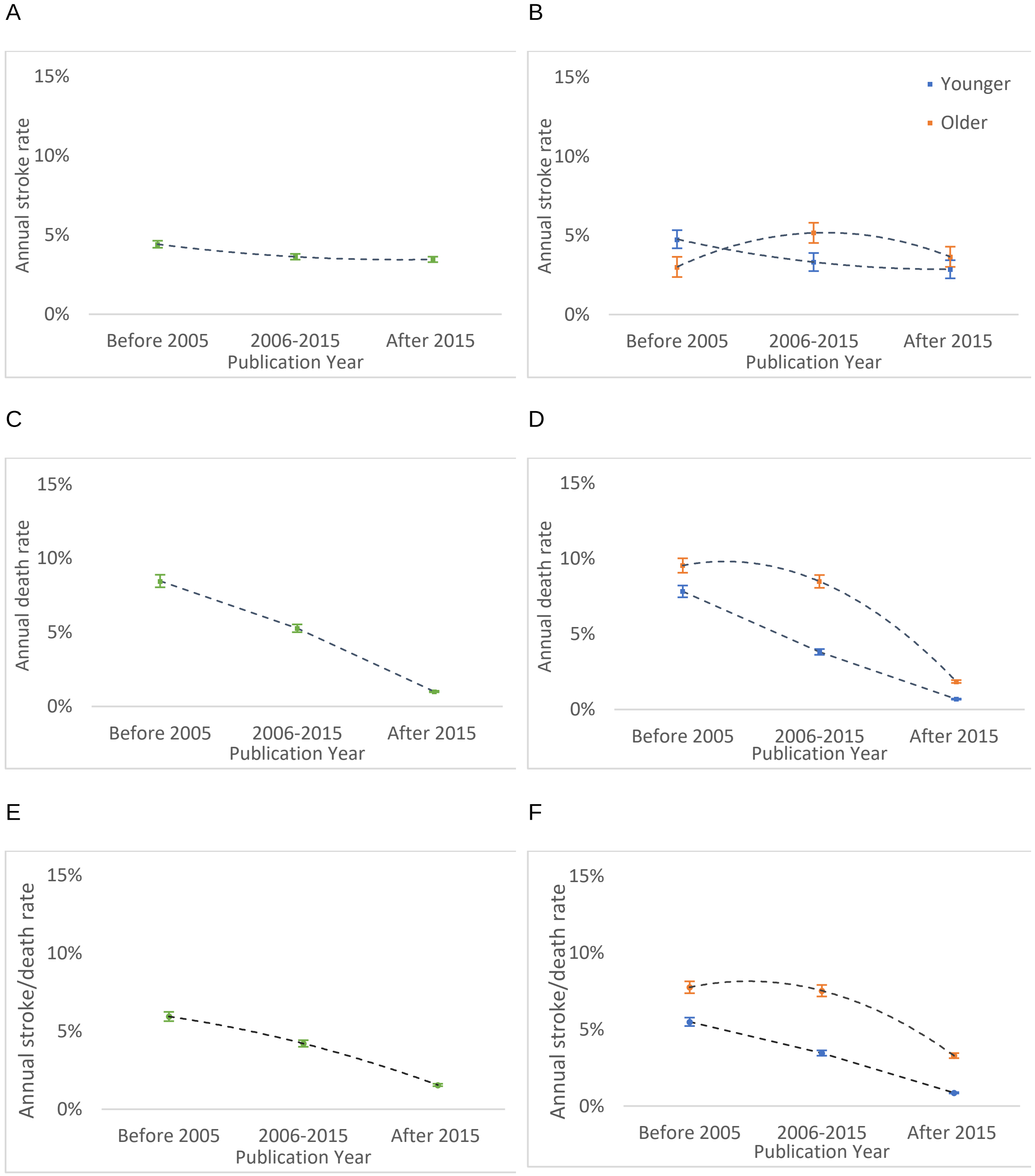

Table S3 Characteristics of the patients included in studies providing individual patient-level dataset

	Stroke					MI					Death				
Variable	Exposed	Unexposed	**OR**	**CI**	**χ2**	Exposed	Unexposed	**OR**	**CI**	**χ2**	Exposed	Unexposed	**OR**	**CI**	**χ2**
**Octogenarian**	33/1043	139/3419	0.70	0.44-1.04	0.09291	25/1043	54/3419	**1.53**	**0.95-2.47**	**0.039865**	16/1043	34/3419	1.55	0.85-2.82	0.0737
**Female sex**	98/2742	71/1720	0.86	0.63-1.18	0.1728	45/2742	34/1720	0.83	0.53-1.30	0.20405	28/2742	22/1720	0.80	0.45-1.40	0.21285
**Hypertension**	104/2642	50/1380	1.09	0.77-1.53	0.3116	50/2642	29/1380	0.90	0.57-1.43	0.32515	32/2642	17/1380	0.98	0.54-1.78	0.47735
**Diabetes**	50/1104	104/2918	1.28	0.91-1.81	0.07745	25/1104	54/2918	1.23	0.76-1.98	0.1993	14/1104	35/2918	1.06	0.57-1.97	0.4297
**Hyperlipidaemia**	7/169	5/187	1.57	0.40-5.05	0.2220	1/169	1/187	1.11	0.07-17.8	0.7248*	4/169	3/187	1.49	0.33-6.78	0.4444*
**Smoking**	12/378	134/3366	0.79	0.43-1.44	0.2093	5/378	73/3366	0.60	0.24-1.15	0.13805	4/378	45/3366	0.79	0.28-2.21	0.4398*
**Coronary artery disease**	35/946	119/3076	0.95	0.65-1.40	0.40645	22/946	57/3076	1.26	0.77-2.07	0.1799	12/946	37/3076	1.06	0.55-2.03	0.4361
**Peripheral arterial disease**	1/59	40/1505	0.63	0.09-4.67	0.5366*	1/59	3/1505	8.63	0.88-84.2	0.1427*					
**Atrial fibrillation**	3/119	18/810	1.14	0.33-3.92	0.5172*										
**Chronic lung disease**	16/427	126/3239	0.96	0.56-1.63	0.44275	14/427	63/3239	1.71	0.95-3.08	0.03545	10/427	32/3239	2.40	1.17-4.92	0.00674
**Chronic kidney disease**	3/71	139/3598	1.10	0.34-3.53	0.5233*						1/71	41/3598	1.24	0.17-9.13	0.5619*
**Baseline antiplatelet**	9/335	2/124	1.68	0.36-7.90	0.3924*						2/335	1/124	0.74	0.07-8.21	0.6122*
**Baseline lipid-lowering**	39/1,464	13/424	0.86	0.46-1.63	0.328	2/1,464	2/424	0.29	0.04-2.06	0.2195*	4/1,464	1/424	1.16	0.13-10.39	0.6864*

Figure S6 Egger Funnel Plot and Forest Plot for assessment of four prospective studies included in the individual patient-level data multivariate analysis, reporting rates of perioperative stroke in patients with symptomatic carotid stenosis stratified by age

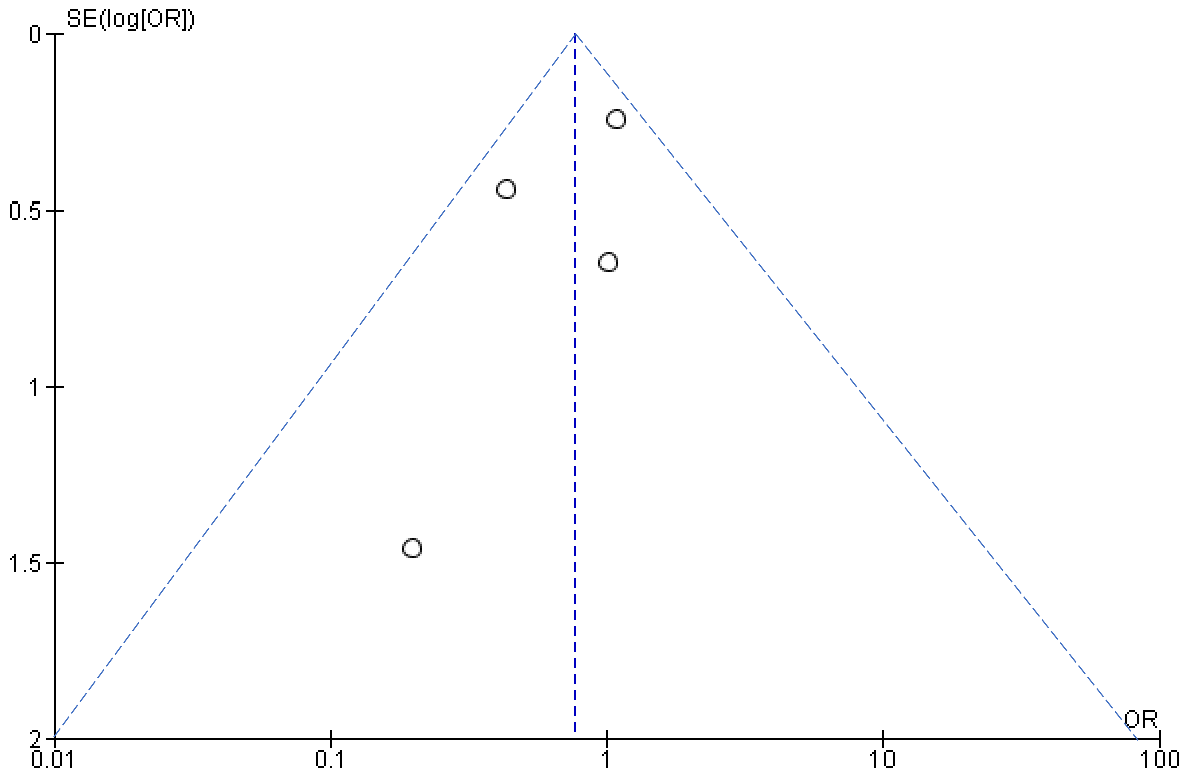

Study or Subgroup	Octogenarian Events	Octogenarian Total	Non-octogenarian Events	Non-octogenarian Total	Weight	Risk Ratio M-H, Random, 95% CI
CHACDP, USA	21	426	92	2033	50.9%	1.09 [0.69, 1.73]
EVA-3S, Fraance	0	52	9	207	4.1%	0.21 [0.01, 3.49]
Thames Valley, UK	6	433	36	1142	28.7%	0.44 [0.19, 1.04]
Umbria, Italy	5	99	4	71	16.4%	0.90 [0.25, 3.22]
**Total (95% CI)**		**1010**		**3453**	**100.0%**	**0.76 [0.42, 1.37]**
Total events	32		141			

Heterogeneity: Tau² = 0.12; Chi² = 4.48, df = 3 (P = 0.21); I² = 33%
Test for overall effect: Z = 0.92 (P = 0.36)

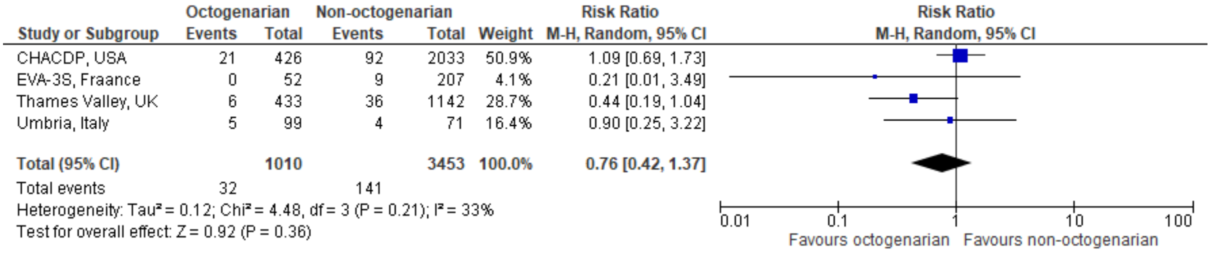

Figure S7 Annual stroke and death rate stratified by prevalence of vascular risk factors

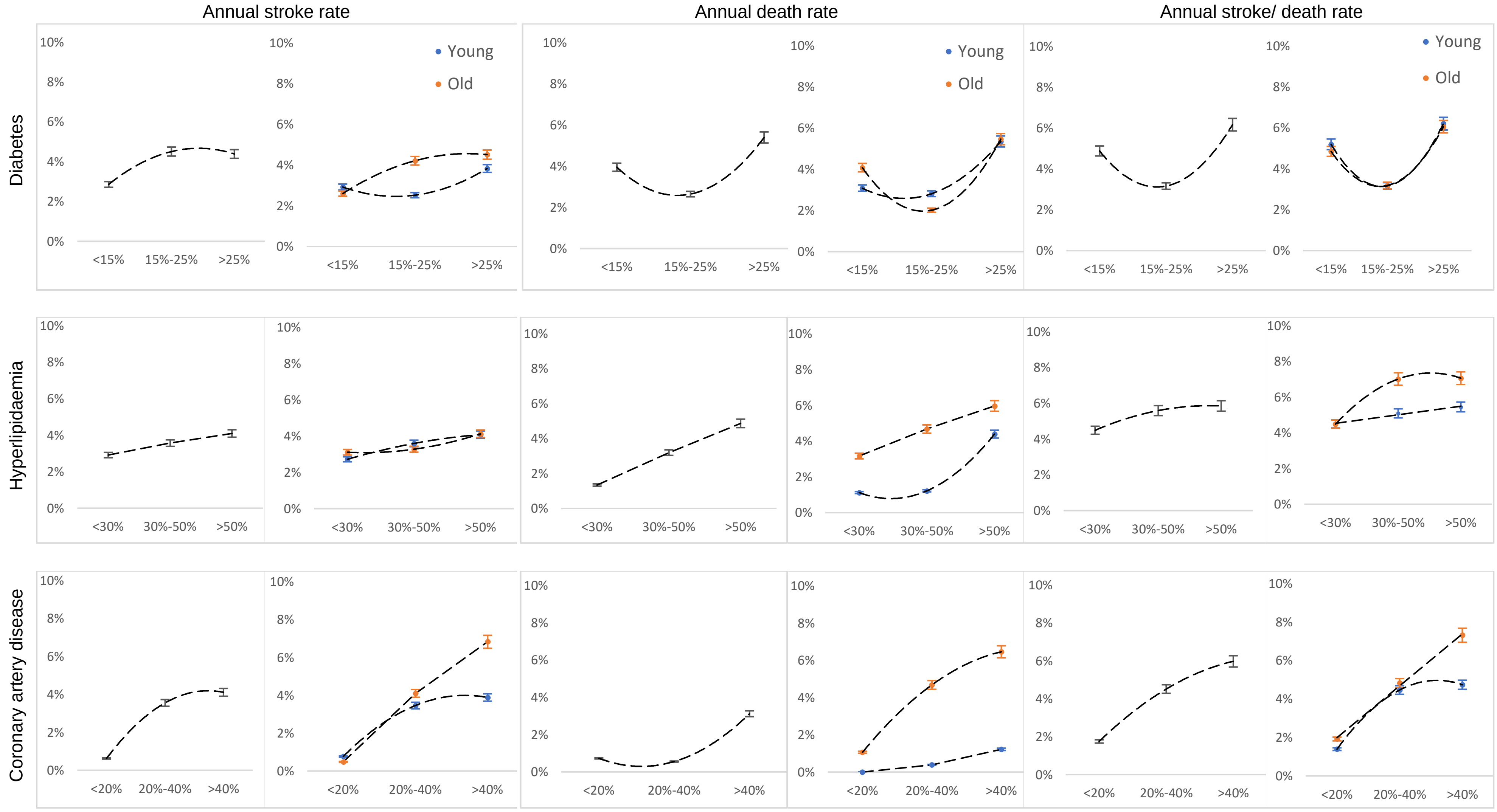

Supplement: Supplementary file 1 [file str-54-457-s001.pdf]
